# Transformer models in cardiovascular medicine: current applications, translational challenges, and future directions

**DOI:** 10.3389/frai.2026.1962796

**Published:** 2026-10-08

**Authors:** Yang Yan, Gaoxiang Li, Taiwei Chen, Jinchuan Yan, Chen Wang, Ancai Yuan, Jiawen Zhang, Jun Pu

**Affiliations:** 1 Department of Cardiology, State Key Laboratory for Oncogenes and Related Genes, Shanghai Cancer Institute, Renji Hospital, School of Medicine, Shanghai Jiao Tong University, Shanghai, China; 2 Department of Cardiology, Zhenjiang Ruikang Hospital, Zhenjiang, Jiangsu, China; 3 Department of Dermatology, Huashan Hospital, Fudan University, Shanghai, China

**Keywords:** artificial intelligence, cardiovascular diseases, current applications, deep learning, future directions, Transformer models, translational challenges

## Abstract

Cardiovascular diseases remain a leading cause of mortality and morbidity worldwide, highlighting the need for improved early detection, accurate diagnosis, and personalized therapeutic strategies. With advances in artificial intelligence (AI), Transformer-based models have attracted increasing interest because they can model long-range dependencies, support large-scale pre-training, and integrate heterogeneous data. Originally developed for natural language processing, Transformers have been extended to medical image analysis, physiological signal interpretation, and clinical text mining. In cardiovascular medicine, these models have been applied to electrocardiogram interpretation, cardiac imaging, electronic health record analysis, multimodal data integration, digital-twin development, and large language model-enabled workflows. Although many studies report promising performance, clinical value remains contingent on data quality, external validation, calibration, workflow integration, and trustworthy governance. This narrative review provides a focused overview of Transformer-based models in cardiovascular medicine, critically evaluates their translational limitations, and outlines priorities for clinically useful and responsible cardiovascular AI.

## Graphical Abstract

**Figure:**
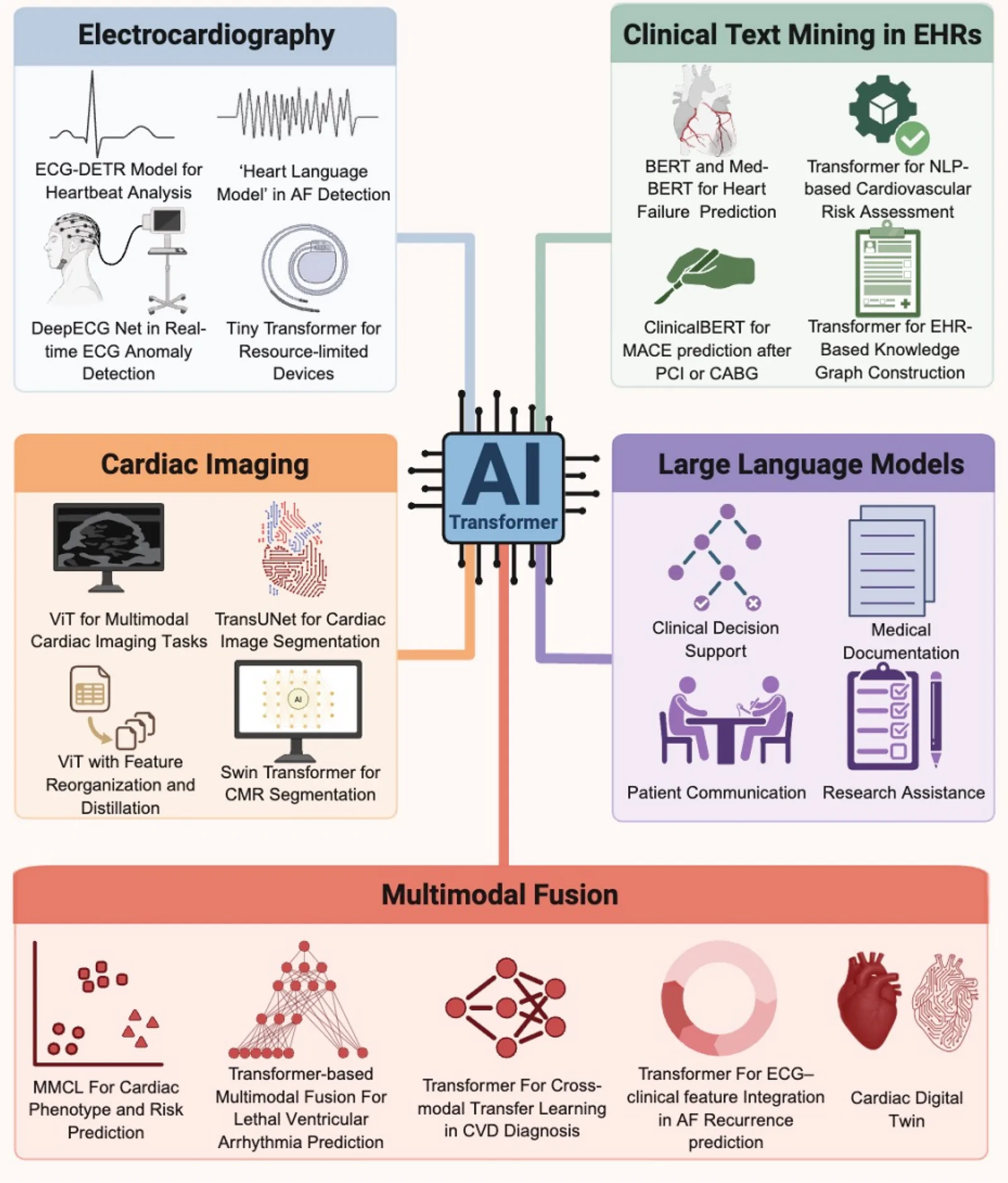
Created by the authors using BioRender.com.

## 1 Introduction

Cardiovascular diseases (CVDs) remain the leading cause of mortality worldwide, accounting for an estimated 19.2 million deaths in 2023 (Global Burden of Cardiovascular Diseases and Risks 2023 Collaborators, 2025). Despite advances in pharmacological therapies, imaging technologies, and preventive strategies, the early identification, accurate risk stratification, and effective management of CVDs continue to pose substantial challenges in clinical practice and public health (Luo Y. et al., 2024). With the exponential growth and increasing heterogeneity of healthcare data, these challenges have become even more pronounced. The rapid expansion of multimodal data sources, including electrocardiography (ECG), cardiac imaging, and electronic health records (EHRs), has made it increasingly difficult for clinicians to interpret vast amounts of information efficiently and accurately, thereby complicating clinical decision-making (Esteva et al., 2019; Noor et al., 2025; Vaid et al., 2023).

In recent years, artificial intelligence (AI), particularly deep learning, has demonstrated remarkable potential in advancing medical diagnostics, prognostication, and clinical decision support (Esteva et al., 2019; Topol, 2019). Among various deep learning architectures, the Transformer has emerged as a pivotal innovation. Initially designed for natural language processing (NLP) tasks, the Transformer leverages a self-attention mechanism that enables it to effectively capture long-range dependencies (Vaswani et al., 2017). Compared with traditional recurrent neural networks (RNNs) and convolutional neural networks (CNNs), this architecture offers significant advantages in scalability, parallel computing capability, and processing of high-dimensional biomedical data (Nerella et al., 2024; Chen J. et al., 2023). Owing to these advantages, Transformer models have been increasingly investigated across a wide range of medical domains, notably in cardiovascular medicine.

Unlike previous reviews that have primarily focused on broad applications of artificial intelligence or individual Transformer-based techniques in cardiovascular medicine, this review provides an integrated, task-oriented synthesis spanning ECG analysis, cardiac imaging, EHR mining, multimodal learning, cardiac digital twins, and large language model–enabled workflows. Importantly, we distinguish Transformer architectures from foundation models and LLMs, and place greater emphasis on the level of validation, generalizability, and evidence of clinical utility rather than benchmark performance alone. We further examine translational challenges related to calibration, external validation, human–AI interaction, governance, and lifecycle monitoring. By combining technical developments with a clinical translation framework, this review aims to clarify where Transformer-based approaches currently add value, where evidence remains insufficient, and what is required for responsible implementation in cardiovascular care.

### 1.1 Literature search and study selection

To improve transparency while retaining a narrative-review design, we performed a structured literature identification and update using PubMed, Embase, Web of Science, Scopus, IEEE Xplore for publications from January 1, 2017 through September 1, 2026. Search concepts combined model terms (“Transformer,” “attention,” “BERT,” “vision transformer,” “foundation model,” “large language model,” “multimodal,” or “diffusion transformer”) with cardiovascular terms (“cardiovascular,” “cardiology,” “ECG,” “electrocardiogram,” “echocardiography,” “cardiac magnetic resonance,” “cardiac CT,” “electronic health record,” or “digital twin”). We prioritized English-language original studies that developed, validated, or applied Transformer-based or Transformer-backbone methods to cardiovascular data or clinical tasks, and retained landmark architecture papers, reporting guidelines, and key reviews for context. Reference lists of relevant reviews and included studies were hand-screened. When a peer-reviewed version of record was available, it was preferred. Conference papers and publicly archived preprints were retained selectively for rapidly evolving or proof-of-concept topics when no version of record was identified; these are explicitly labeled and were not treated as equivalent to externally validated clinical evidence. Duplicates, non-cardiovascular applications, and non-Transformer studies were excluded unless used as comparators or background. Study selection was not performed in duplicate and no formal risk-of-bias tool or meta-analysis was applied; therefore, the literature set should not be interpreted as exhaustive. Generative AI was not used to autonomously determine study eligibility, and all factual statements and bibliographic details added during revision were manually checked against source publications.

## 2 Overview of the Transformer architecture

The Transformer architecture, introduced by Vaswani et al. (2017), was initially developed for NLP. By introducing the self-attention mechanism, the Transformer fundamentally reshaped the processing of sequential data and has been widely recognized as a milestone innovation in the evolution of deep learning (Hochreiter and Schmidhuber, 1997; Lecun et al., 1998).

The original Transformer was formulated as an encoder–decoder architecture for sequence transduction (Vaswani et al., 2017). Within the encoder–decoder framework, the model first converts tokens (the smallest textual units) into vectors (embeddings) and then learns their contextual relationships through multiple layers of self-attention and feed-forward networks, enabling each token to integrate global information (Vaswani et al., 2017). Because self-attention itself does not encode sequence order, positional encodings are added to the input embeddings. Each Transformer layer further incorporates multi-head attention, position-wise feed-forward networks, residual connections, and layer normalization (Vaswani et al., 2017). Multi-head attention allows the model to learn complementary relationships across multiple representation subspaces. These components collectively support flexible contextual representation across diverse tasks (Devlin et al., 2019; Brown et al., 2020).

The core innovation of the Transformer lies in the self-attention mechanism, which functions as a form of “focused search.” In standard full self-attention, when processing a particular element in the input (such as text, images, or physiological signals), the model can attend to all positions in the sequence and adaptively assign greater weight to the most relevant components while attenuating the contribution of less informative content (Vaswani et al., 2017; Devlin et al., 2019). Restricted, windowed, or sparse-attention variants may instead attend only to subsets of the input. This enables the Transformer to capture both local details and global information within a single computation, while allowing greater parallelization than recurrent computation (Vaswani et al., 2017; Dosovitskiy et al., 2020). However, standard full self-attention has quadratic computational and memory complexity with respect to sequence length, and therefore is not universally more computationally efficient than RNNs or CNNs. In contrast, traditional recurrent neural networks (RNNs) process information sequentially, similar to passing a relay baton, which not only constrains efficiency but also can make long-range dependencies more difficult to model (Hochreiter and Schmidhuber, 1997). Another classical architecture, convolutional neural networks (CNNs), constructs hierarchical representations through the progressive integration of local features, resembling “building blocks.” This approach excels at capturing local contextual patterns but remains limited in modeling long-range dependencies within the input (Lecun et al., 1998; Baker et al., 2018). Global information in CNNs is generally accumulated through deeper layers, larger receptive fields, or specifically designed architectural modules (Figure 1) (Rezvani et al., 2023; Hou and Li, 2025).

**Figure 1:**
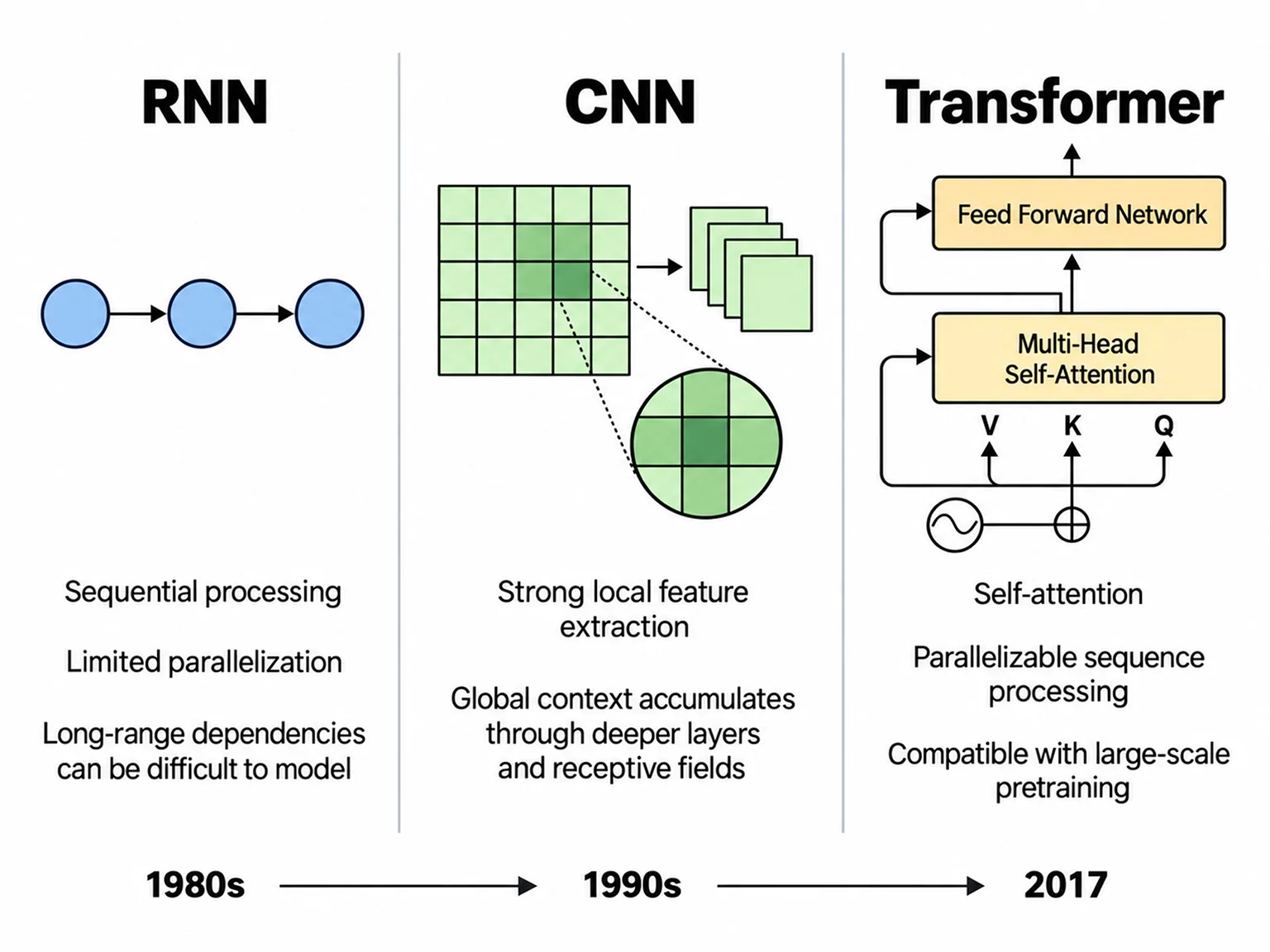
Schematic comparison of recurrent neural network (RNN), convolutional neural network (CNN), and Transformer architectures. Created by the authors using BioRender.com, with AI-assisted visual design supported by OpenAI.

Two additional advantages of the Transformer are its remarkable scalability and the pre-training paradigm (Wolf et al., 2019). Importantly, pre-training is a training strategy rather than an inherent property of the Transformer architecture. Transformer models may be trained from scratch or pre-trained on large datasets and subsequently adapted to downstream tasks. It can be pre-trained on massive unlabeled datasets to learn general feature representations and subsequently fine-tuned for diverse downstream tasks (Devlin et al., 2019). This paradigm not only significantly reduces dependence on annotated data but also greatly enhances generalization across tasks and domains when sufficient and appropriately matched pre-training data are available (Wolf et al., 2019; Alzubaidi et al., 2023).

Leveraging this flexibility, the Transformer has rapidly expanded beyond natural language processing to computer vision, multimodal learning, and other fields (Esteva et al., 2019; Dosovitskiy et al., 2020). More importantly, its ability to integrate heterogeneous data modalities has laid the foundation for emerging applications, including medical imaging and clinical data analysis, which are further discussed in subsequent sections. For consistency throughout this review, Transformer architectures are broadly categorized as encoder-only models (e.g., BERT), decoder-only autoregressive models (e.g., GPT-family models), and encoder–decoder models, while hybrid CNN–Transformer and multimodal/cross-attention architectures are treated as architectural extensions (Vaswani et al., 2017; Devlin et al., 2019; Brown et al., 2020). Foundation models and LLMs are distinguished by their training scale and functional scope rather than considered separate Transformer architectures.

## 3 Current applications of Transformer models in cardiovascular medicine

The available studies vary considerably in study design, dataset characteristics, sample size, and validation strategy, which limits direct comparison of reported performance. In particular, strong results obtained from retrospective benchmark datasets should not be interpreted as equivalent to external validation or demonstrated clinical utility. Table 1 therefore provides a structured comparison of representative studies according to cardiovascular task, publication status, Transformer architecture, dataset, sample size, split unit, validation strategy, comparator(s), performance metrics, and key limitations.

**Table 1:** Representative Transformer-based studies in cardiovascular medicine: tasks, datasets, validation, performance, and limitations.

Cardiovascular task	Representative study	Publication status	Transformer architecture	Dataset	Sample size	Split unit	Validation strategy	Comparator(s)	Performance metrics	Key limitations
ECG arrhythmia detection	Hu et al., 2022	Peer-reviewed journal article	ECG-DETR; end-to-end Transformer detector for simultaneous heartbeat localization and classification	MIT-BIH Arrhythmia Database; MIT-BIH Atrial Fibrillation Database (AFDB)	MIT-BIH: 48 recordings from 47 subjects; AFDB: 25 long-term recordings	Not clearly reported as patient-disjoint	10-fold cross-validation; evaluated on 8-, 4-, and 2-label arrhythmia tasks	Prior published heartbeat segmentation/ classification methods; no single standardized head-to-head comparator	Accuracy: 99.12% (8-label), 99.49% (4-label), 99.23% (2-label)	Public benchmark datasets only; no independent clinical-site or prospective validation; patient-level independence of cross-validation should be interpreted cautiously
AF detection/ECG foundation model	Tudjarski et al., 2025	Peer-reviewed journal article	Heart language model; bidirectional Transformer foundation model over tokenized heartbeat/RR-interval annotations	12 public ECG benchmark databases for pre-training; MITDB for fine-tuning; AFDB, LTAFDB and other databases for testing	FDB pre-training corpus: 22,858,158 original annotated heartbeats (51,590,827 after database repetition); MITDB: 48 recordings from 47 subjects	Inter-patient/cross-database evaluation; MITDB used for fine-tuning and separate databases used for testing	Cross-database, inter-patient evaluation; fine-tuned on MITDB and tested on unseen ECG databases	Pereira et al. and Jahan et al. AF-detection methods under cross-database inter-patient evaluation; direct comparison limited by different training settings	Duration-based F1: 91.97% (AFDB), 93.33% (LTAFDB), 87.37% (TDB)	Depends on heartbeat/QRS-derived tokenization and benchmark annotations; public-dataset setting; no prospective real-world deployment study
Resource-constrained arrhythmia classification	Busia et al., 2025	Peer-reviewed journal article	Tiny Transformer (~6.6k parameters) with RR-interval input; 8-bit quantized deployment	MIT-BIH Arrhythmia Database; MIT-BIH Noise Stress Test data for robustness experiments	48 recordings from 47 subjects (~100,000 annotated beats)	Heartbeat-level intra-patient split; not patient-disjoint	Random 7:1:2 train/validation/ test heartbeat split with five-fold cross-validation; clean and noise-corrupted testing	Published ViT, Transformer, MLP, and CNN MIT-BIH models; state-of-the-art ViT used as a main efficiency/accuracy reference	8-bit accuracy 98.97% (clean) and 98.36% (noise-mix); 4.28 ms and 0.09 mJ per inference on GAP9	Intra-patient evaluation can overestimate patient-level generalization; marked class imbalance and poor performance for very rare classes; single main benchmark dataset
Cardiac MRI segmentation	Zhao et al., 2022	Peer-reviewed journal article	nn-TransUNet: Vision Transformer + convolutional encoder with convolutional decoder and automated experiment planning	ACDC; MSD Task02 Heart; MyoPS 2020	ACDC: 150 patients/300 cine-MRIs overall; experiment used 180 cases from 90 patients for development (36 validation cases) and 20 cases from 10 patients for testing. MSD02: 30 MRIs (20/5/5). MyoPS: the article gives both 30 and 25 cases; reported split 15/5/5	ACDC: patient-level train/test split, with validation selected from development MRI cases; MSD02/MyoPS: case-level	Fixed internal splits: ACDC full-data experiment 144/36/20 cases (train/validation/ test); MSD02 20/5/5; MyoPS 15/5/5	R50 U-Net, R50 Att-UNet, ViT, nnU-Net, and TransUNet	ACDC: mean Dice 93.6%; Hausdorff distance 9.1 mm; Jaccard 88.3%	Small fixed test sets; no independent hospital-level clinical validation; performance declines with reduced training data
Coronary CTA plaque segmentation/ detection	Alvén et al., 2025	Peer-reviewed journal article	PlaqueViT; customized 3D Vision Transformer deep ensemble (k = 6) for voxelwise classification	SCAPIS CCTA cohort; independent Linköping University Hospital external set	463 development, 123 internal test, 65 reader-agreement cases, 28 external cases; separate CAD-detection set *n* = 684	Subject-level	Random 80/20 development/ internal-test split; six-fold cross-validation within development data; separate reader-agreement analysis and independent external validation	Expert-reader manual annotations and reader-agreement benchmark; no alternative-model head-to-head comparator	Plaque Dice 0.55; average surface distance 0.98 mm; ICC 0.93. CAD detection: 88% agreement (97% sensitivity, 76% specificity) in internal test	External validation set was small (*n* = 28); poor-quality scans were excluded; endpoints were segmentation/ plaque detection rather than patient outcomes
EHR cardiovascular risk-factor extraction	Houssein et al., 2023	Peer-reviewed journal article	Stacked BERT + CharacterBERT contextual embeddings with an RNN sequence classifier	2014 i2b2/UTHealth heart-disease risk-factor challenge corpus	1,304 clinical records from 296 patients with diabetes; 790 training and 514 test records	Record-level; patient-disjointness not clearly reported	Organizer-defined 60/40 training/test split; challenge evaluation script	Alternative embedding/model configurations developed in the study and prior 2014 i2b2/UTHealth challenge systems	Overall micro-F1 93.66%; macro-F1 ~70%	Curated, manually annotated single shared-task corpus; rare temporal labels and class imbalance; no external institutional validation
Six-month mortality prediction from EHRs	Antikainen et al., 2023	Peer-reviewed journal article	BERT and XLNet applied to longitudinal heterogeneous EHR event sequences	Tays Heart Hospital/ Pirkanmaa EHR, KARDIO registry, Finnish mortality registry	57,377 eligible adults; model evaluation on 23,542 patients (9,640 train, 2,420 validation, 11,482 held-out test)	Patient-level	Development set split 80/20 into training/ validation; stratified five-fold cross-validation within development data; final models evaluated on a separate held-out test set, with training repeated across five random initializations	BERT vs. XLNet (head-to-head within the same study)	Mean AUC: 75.5% (BERT), 76.0% (XLNet); XLNet had higher recall	Single healthcare system; substantial class imbalance and low positive predictive value; no independent external-site validation
AF recurrence after catheter ablation	Qiu et al., 2024	Peer-reviewed journal article	End-to-end Transformer ECG encoder with multimodal fusion of ECG and clinical variables	Single-center AF ablation cohort with standard 10-s surface ECGs	920 patients; 253 recurrences (27.5%)	Patient-level; patients and ECGs assigned together	Random 7:1:2 training/internal-validation/test split; no external validation cohort	ECG-only and clinical-only Transformer models; CHA2DS2-VASc, APPLE, and DR-FLASH clinical risk scores	Multimodal model: AUROC 0.899; sensitivity 81.1%; specificity 81.7%; accuracy 81.5%; F1 71.7%	Single-center retrospective design; no external validation; some ECGs obtained after ablation; limited interpretability
Lethal ventricular arrhythmia risk in HCM	Lai C. et al., 2025	Peer-reviewed journal article	MAARS: 3D-ViT for LGE-CMR + FNN branches for EHR/report variables + Multimodal Bottleneck Transformer fusion	Johns Hopkins HCM registry (internal); Sanger Heart and Vascular Institute HCM registry (external)	553 internal patients (19 events); 286 external patients (25 events)	Patient-level	Patient-level five-fold internal cross-validation plus independent external healthcare-system validation	2020 ACC/AHA HCM guideline, 2023 ESC cardiomyopathy guideline, and HCM Risk-SCD calculator	AUROC 0.89 (95% CI 0.79–0.94) internally and 0.81 (0.69–0.93) externally; internal balanced accuracy 0.80	Rare endpoint and small event counts; modest external cohort; some external covariates missing/imputed; retrospective registry design
Cardiac anatomical digital-twin reconstruction	Yu J. et al., 2025	Peer-reviewed conference paper (MICCAI 2025)	UltraTwin: conditional Diffusion Transformer with coarse-to-fine refinement and topology-aware implicit autoencoder	Prospectively collected multicenter (nine hospitals) paired echocardiography-CT dataset	891 paired cases/patients (96 ECG-gated, 795 non-strict pairs); 130 strictly paired reconstruction samples split 96/10/24 for train/validation/ test; additional cases used for pre-training	Patient-level for the strictly paired reconstruction samples	96/10/24 train/validation/ test split for 130 strictly paired samples; additional cases used for pre-training; no independent external test cohort	E-Pix2Vox++, GARNet, UMIFormer, LRGT, and internal component ablations	With pre-training: mean Dice 77.27%; mean Hausdorff distance 5.07; mean volumetric error 22.56 mL	Strictly paired test set was very small (*n* = 24); reliance on pseudo-paired pre-training; validates anatomical reconstruction rather than physiological prediction or clinical benefit
Automated HEART score/60-day MACE assessment	Wright et al., 2025	Peer-reviewed journal article	GPT-4 with an iteratively developed prompt applied to de-identified chart documentation	Chest-pain observation-unit EHR cohort	601 participants; 50 for prompt development and 551 for evaluation	Participant-level	Retrospective held-out evaluation; disagreements adjudicated by an emergency physician blinded to outcomes	APP-assigned and physician-adjudicated HEART scores; 60-day MACE outcome	Weighted kappa vs. adjudicator 0.67 (APP vs. adjudicator 0.66); no significant difference in 60-day MACE discrimination; GPT-4 mean HEART score 5.06 vs. 4.69 adjudicated	Single-center retrospective evaluation; systematic score inflation; prompt dependence/model-version reproducibility; no prospective workflow-impact validation
Myocarditis decision support from CMR reports	Kaya et al., 2024	Peer-reviewed journal article	GPT-4 LLM integrating CMR report text with clinical and laboratory information	CMR reports from eight tertiary centers	396 patients with suspected myocarditis	No model-training split; evaluation cohort only	Multicenter retrospective comparison against consensus of two board-certified CMR radiologists; comparison with readers of different experience	Radiologists with 1, 2, and 4 years of CMR experience; consensus of two board-certified CMR radiologists as reference standard	Accuracy 83%; sensitivity 90%; specificity 78%	Report-based rather than raw-image interpretation; lower accuracy than more experienced readers; no prospective clinical workflow or outcome validation
Echocardiography report summarization	Chao et al., 2025	Peer-reviewed journal article	EchoGPT: QLoRA fine-tuned Llama-2; compared with other open-source LLMs	Mayo development data; Mayo Arizona geographic validation; MIMIC-EchoNotes external dataset	97,506 development reports from 71,717 patients (95,506 train, 1,000 validation, 1,000 internal test); Arizona *n* = 1,000; MIMIC-EchoNotes *n* = 1,000	Report-level; patient-disjointness within the development split not clearly reported	Internal split 95,506/1,000/1,000; external validation: Arizona *n* = 1,000 and MIMIC-EchoNotes *n* = 1,000; blinded review of 30 randomly selected cases by four echo-board-certified cardiologists	Llama-2, MedAlpaca, Zephyr, and Flan-T5; cardiologist-generated impressions and blinded cardiologist review	Internal: BLEU 45.9, METEOR 62.4, ROUGE-L 55.7, BERTScore 91.6, RadGraph F1 47.7; performance decreased on MIMIC-EchoNotes	Performance degraded with site/style shift; factual/numerical hallucination risk remains; automatic text metrics incompletely reflect clinical correctness; physician review is still required

3D, three-dimensional; ACDC, Automated Cardiac Diagnosis Challenge; AF, atrial fibrillation; AFDB, MIT-BIH Atrial Fibrillation Database; APP, advanced practice provider; AUC, area under the curve; AUROC, area under the receiver operating characteristic curve; BERT, Bidirectional Encoder Representations from Transformers; BLEU, Bilingual Evaluation Understudy; CAD, coronary artery disease; CCTA, coronary computed tomography angiography; CI, confidence interval; CMR, cardiovascular magnetic resonance; CT, computed tomography; CTA, computed tomography angiography; DETR, DEtection TRansformer; ECG, electrocardiogram; EHR, electronic health record; F1 score, harmonic mean of precision and recall; FNN, feed-forward neural network; GPT-4, Generative Pre-trained Transformer 4; HCM, hypertrophic cardiomyopathy; HEART, History, ECG, Age, Risk factors, and Troponin; i2b2, Informatics for Integrating Biology and the Bedside; ICC, intraclass correlation coefficient; LGE, late gadolinium enhancement; LLM, large language model; LTAFDB, Long-Term Atrial Fibrillation Database; MAARS, Multimodal Artificial intelligence for ventricular Arrhythmia Risk Stratification; MACE, major adverse cardiovascular events; METEOR, Metric for Evaluation of Translation with Explicit ORdering; MIMIC, Medical Information Mart for Intensive Care; MIT-BIH, Massachusetts Institute of Technology-Beth Israel Hospital; MITDB, MIT-BIH Arrhythmia Database; MRI, magnetic resonance imaging; MSD, Medical Segmentation Decathlon; MyoPS, myocardial pathology segmentation; QLoRA, Quantized Low-Rank Adaptation; QRS, QRS complex; RNN, recurrent neural network; ROUGE-L, Recall-Oriented Understudy for Gisting Evaluation-Longest Common Subsequence; RR interval, interval between consecutive R waves; SCAPIS, Swedish CArdioPulmonary bioImage Study; TDB, Testing ECG DataBase collection; UTHealth, University of Texas Health Science Center at Houston; ViT, Vision Transformer.; ACC/AHA, American College of Cardiology/American Heart Association; ESC, European Society of Cardiology; MLP, multilayer perceptron.

### 3.1 Electrocardiography

Electrocardiography (ECG) is the most widely used non-invasive method for diagnosing and monitoring cardiovascular diseases. Traditional deep learning models, mainly RNNs for sequential data and CNNs for extracting local signal features, have shown encouraging performance in arrhythmia classification and atrial fibrillation (AF) detection (Murat et al., 2021; Ansari et al., 2023). However, these models remain limited in analyzing complex or long-duration ECG signals (Ikram et al., 2025).

In contrast, self-attention enables Transformer models to capture local waveform features while modeling longer-range rhythm dependencies, which may be useful for complex or long-duration ECG (e.g., abnormal short waves and rhythm patterns across multiple heartbeats) analysis. For example, Hu et al. (2022) proposed the ECG-DETR model, which directly predicts both the position and type of heartbeats from continuous single-lead ECG data, reporting overall accuracies of 99.12%, 99.49%, and 99.23% across three arrhythmia-detection tasks using stratified 10-fold cross-validation. These results were comparable to previous methods; however, the cross-validation design should not be interpreted as independent patient-level validation. Recently, researchers proposed a “heart language model,” which treats heartbeat sequences as “tokens,” similar to words within a sentence. By fine-tuning a pre-trained Transformer, the model reached an F1 score (the harmonic mean of precision and recall) above 93% in AF detection, highlighting the potential of pre-trained Transformer models for ECG analysis (Tudjarski et al., 2025). F1 scores should be interpreted in the context of class prevalence, decision threshold, and the relative clinical consequences of false-positive and false-negative predictions. Hybrid architectures, such as DeepECG-Net, combine CNN for local feature extraction with Transformer for global dependency modeling. DeepECG-Net reported 98.2% precision and a 97.5% F1 score using a 70%/15%/15% sample split; however, the absence of clearly patient-disjoint validation and limited class-wise reporting restrict conclusions regarding generalizability and performance in minority arrhythmia classes (Alghieth, 2025). For resource-limited scenarios, the Tiny Transformer contains only about 6,000 parameters, and reported 98.97% accuracy on the MIT-BIH dataset under an intra-patient evaluation setting. Class-wise sensitivity and precision decreased particularly for minority S and F classes under noise. Deployment on the GAP9 processor achieved 4.28 ms inference time and 0.09 mJ per inference, supporting low-power engineering feasibility, although this does not constitute clinical wearable validation (Busia et al., 2025).

Across the reviewed ECG studies, pure Transformers, CNN-Transformer hybrids, and pre-trained token-based models have achieved strong benchmark performance in arrhythmia detection, AF screening, and long-sequence analysis (Ikram et al., 2025; Hu et al., 2022; Tudjarski et al., 2025; Alghieth, 2025; Busia et al., 2025). However, direct comparisons remain dataset-dependent, and future work should prioritize external validation, label quality, calibration, and real-time evaluation in wearable devices.

### 3.2 Cardiac imaging

Cardiac imaging, including cardiac magnetic resonance (CMR), cardiac computed tomography (CT), and echocardiography, plays an important role in identifying structural and functional abnormalities of the heart. CNNs, which extract local features from images, have demonstrated good performance in segmentation and classification tasks (Kwan et al., 2021). However, constrained by their limited receptive field, CNNs struggle to capture global spatial representation and may be less direct in modeling long-range spatial dependencies; consistency across slices or temporal frames generally requires dedicated volumetric or spatiotemporal modeling (Khan et al., 2023; Lara Hernandez et al., 2021).

The Vision Transformer (ViT), introduced by (Dosovitskiy et al. 2020), adapted a standard Transformer encoder to sequences of image patches and demonstrated the feasibility of a largely attention-based architecture for image classification. ViT achieved competitive performance relative to CNN-based approaches under the experimental settings evaluated in the original study (Dosovitskiy et al., 2020). In the field of cardiac imaging, ViT has been successfully applied across a variety of tasks, such as delineation of ventricular and atrial structures in CMR segmentation, coronary vessel and plaque segmentation in coronary CT angiography, and view classification and chamber segmentation in echocardiography (Zhao et al., 2022; Chen Z. et al., 2023; Alvén et al., 2025; Liao et al., 2023; Zhang et al., 2026). Moreover, hybrid frameworks such as TransUNet, which integrate the local feature extraction capability of CNNs with ViT to capture long-range dependencies and global contextual relationships, have demonstrated outstanding performance in cardiac image segmentation and laid the foundation for subsequent Transformer variants tailored to medical imaging (Chen et al., 2024). Unlike TransUNet, which focuses on architectural fusion, Vision Transformer with Feature Reorganization and Distillation (ViT-FRD) adopts a knowledge-transfer paradigm in which a CNN serves as the teacher and a ViT as the student. Through feature reorganization and knowledge distillation, ViT-FRD reported strong segmentation performance on two CMR datasets (Fan et al., 2023).

Following ViT, Liu et al. (2021) proposed the Shifted Window Transformer (Swin Transformer), which preserves the advantages of the self-attention mechanism while incorporating hierarchical representations and a shifted-window strategy. The window-based attention design reduces computational complexity with respect to image size, while shifted windows enable information exchange across neighboring windows (Liu et al., 2021). Swin-Unet subsequently adapted this hierarchical architecture to medical image segmentation (Cao et al., 2023). In cardiac MRI segmentation, (Yang et al. 2024) reported a Dice coefficient of 92.28% on the ACDC dataset using a fusion-attention Swin Transformer, while Zhang L. et al. (2025) reported a Dice coefficient of 90.15% on ACDC using FE-SwinUper. These results represent study-specific benchmark performance and do not establish superior cross-slice or cine consistency.

Collectively, ViT and variants such as TransUNet, ViT-FRD, and Swin Transformer have shown promising performance in cardiac imaging, particularly for segmentation. However, cross-dataset and prospective clinical validation remain limited, and reported superiority should be interpreted in the context of dataset size, benchmark selection, and study design (Khan et al., 2023; Lara Hernandez et al., 2021; Fan et al., 2023; Yang et al., 2024; Zhang L. et al., 2025).

### 3.3 Clinical text mining in electronic health records (EHRs)

Electronic health records (EHRs) contain a wealth of clinical information, such as medical history, laboratory test results, medication prescriptions, imaging reports, and discharge summaries, serving as an essential resource for clinical decision-making and cardiovascular research (Jensen et al., 2012). However, these comprehensive data are often highly unstructured and heterogeneous in format, posing significant challenges for traditional RNN- and CNN-based models (Hossain et al., 2023; Kim M. K. et al., 2024). Previous methods primarily relied on rule-based systems, traditional machine learning methods (e.g., support vector machines, logistic regression, conditional random fields), and early deep learning architectures (Yao et al., 2019; Ayala Solares et al., 2020; Percha, 2021). Nevertheless, such methods demand extensive manual annotation, exhibit limitations in capturing long-range dependencies, and generalize poorly across institutions and patient populations, thereby constraining the extraction of latent clinical value from EHRs (Spasic and Nenadic, 2020; Guo et al., 2021; Yang et al., 2022).

The introduction of Transformers has greatly advanced clinical text analysis. The BERT (Bidirectional Encoder Representations from Transformers), introduced by Google in 2018, is a bidirectional pre-trained language model built on the Transformer architecture (Devlin et al., 2019). By leveraging large-scale pre-training and task-specific fine-tuning, BERT generates more accurate contextual representations, reshaping the landscape of natural language processing (Devlin et al., 2019). BioBERT and ClinicalBERT extend BERT to biomedical and clinical text (Lee et al., 2020; Huang et al., 2019), whereas Med-BERT adapts the BERT framework to structured longitudinal EHR diagnosis codes for disease prediction (Rasmy et al., 2021). Med-BERT was evaluated for heart failure prediction among patients with diabetes using structured EHR data (Rasmy et al., 2021). Separately, (Kim M. et al. 2024) evaluated an FT-Transformer within a reliable and interpretable AI framework for in-hospital, 6-month, and 12-month mortality after acute myocardial infarction, reporting higher sensitivity but comparable AUROC and F1 scores relative to XGBoost. When dealing with heterogeneous or multi-source EHRs, traditional approaches such as RNNs and CNNs frequently encounter limitations, whereas Transformer models exhibit enhanced representation learning and comprehensive analysis. Houssein et al. (2023) showed that the use of Transformer in NLP can effectively capture information from unstructured clinical texts to identify risk factors for heart disease. (Antikainen et al. 2023) utilized Transformer on multi-source heterogeneous EHRs to predict mortality risk in cardiovascular patients, with improved performance relative to the baseline models evaluated in the same study. Kim et al. (2025) leveraged ClinicalBERT on ICD-10 diagnostic codes from large-scale EMR data to predict 1-year major adverse cardiovascular events in patients undergoing percutaneous coronary intervention (PCI) or coronary artery bypass grafting (CABG). Moreover, Transformers have also been applied to construct EHR-based knowledge graphs, including those for heart failure and cardiovascular pharmacotherapy, thereby offering novel tools to advance precision medicine and clinical decision-making (Xu et al., 2024; Cui et al., 2025).

Overall, with strong capabilities in modeling heterogeneous and multi-source EHRs, Transformer models demonstrate unique advantages in risk prediction, clinical decision support, and clinical knowledge graph construction, indicating their increasing importance in cardiovascular medicine.

### 3.4 Multimodal fusion in cardiovascular medicine

Clinical data in cardiovascular medicine are highly complex and derived from diverse sources, including medical imaging (e.g., CMR, CT, and echocardiography), physiological signals (e.g., ECG, blood pressure, and hemodynamic parameters), EHRs, and multi-omics data (e.g., genomics, transcriptomics, and proteomics). Cardiovascular diseases often involve concurrent structural remodeling, functional abnormalities, and molecular dysregulation, making it difficult for a single-modality examination to comprehensively depict the underlying pathophysiological processes (Cohn et al., 2000; Schirone et al., 2017; Daubert et al., 2021). Consequently, multimodal integration has become a key direction for precise diagnosis and personalized intervention (Acosta et al., 2022). In the past, such integration primarily relied on clinicians' experience and expertise, as well as traditional statistical cardiovascular risk assessment models (such as Framingham, TIMI, and GRACE) (Antman et al., 2000; D'Agostino et al., 2008; Fox et al., 2006). However, these methods have limited capacity to handle heterogeneous multimodal data and achieve genuine information fusion (Acosta et al., 2022; Talha et al., 2024).

In this context, AI is regarded as a key approach to managing the complexity of multimodal medical data. Cross-modal attention mechanism enables models to learn and align associative features across different modalities (Lu et al., 2016, 2019), such as medical imaging, physiological signals, and clinical text, thereby achieving deeper information fusion (Raminedi et al., 2024). Meanwhile, the Query–Key–Value structure (Vaswani et al., 2017) (the core framework of the attention mechanism) underlying the Transformer provides a natural and efficient foundation for implementing cross-modal attention, making the Transformer the most representative and widely adopted model in current multimodal integration research (Acosta et al., 2022; Lymperaiou and Stamou, 2024). For example, Turgut et al. (2025) employed a Transformer-based multimodal contrastive learning (MMCL) framework to transfer CMR-derived information to an ECG encoder through paired ECG–CMR pre-training. After pre-training, downstream inference was performed using ECG alone, enabling cardiovascular risk and phenotype prediction. In addition, Qiu et al. (2024) used a Transformer-based framework in which surface ECG signals and clinical features were encoded separately, and the resulting feature vectors were concatenated after Transformer encoding in a fusion module, reporting an AUROC of 0.899 in predicting atrial fibrillation (AF) recurrence. Moreover, Girlanda et al. (2024) combined self-supervised contrastive learning withmultimodal representation learning, using distinct modality-specific encoders for ECG, CMR imaging, and tabular clinical data, to transfer information from CMR to ECG and clinical data, improving cardiovascular disease prediction relative to the supervised baseline under limited annotated data. Furthermore, Lai C. et al. (2025) proposed a novel Transformer-based neural network that integrates EHRs, clinical notes, echocardiography, radiology reports, and CMR images to accurately predict lethal ventricular arrhythmias.

With the continued advancement of multimodal modeling techniques, the concept of the cardiac digital twin (CDT) has gradually emerged. By integrating imaging data, physiological signals, hematological parameters, and genomic information, the CDT enables individualized virtual heart models that dynamically simulate disease progression and predict therapeutic outcomes, offering new support for precision cardiovascular medicine (Corral-Acero et al., 2020; Sel et al., 2024; Thangaraj et al., 2024). Owing to its exceptional capability in multimodal data fusion, spatiotemporal dependency modeling, and personalized feature extraction, the Transformer may support selected components of CDT construction (Thangaraj et al., 2024). Yu J. et al. (2025) proposed the UltraTwin model based on conditional generation, which employs a Diffusion Transformer (DiT) to reconstruct high-fidelity, patient-specific cardiac anatomical twins from sparse multi-view 2D echocardiographic images, demonstrating potential utility in cardiac chamber volume measurement as an anatomical reconstruction approach rather than a complete functional CDT. Lai Y. et al. (2025) further developed the ECGTwin model, a Transformer-based two-stage diffusion framework capable of generating and reconstructing individualized electrocardiographic signals. ECGTwin showed improved signal quality and downstream task performance relative to the comparison methods evaluated in the original study, highlighting its potential for personalized electrophysiological twin modeling, although clinical validation remains lacking. Meanwhile, Fan et al. established a Transformer-based data-driven surrogate model that efficiently simulates left ventricular dynamics and global hemodynamics. Compared with traditional finite element simulations, this model achieved over a 40,000-fold acceleration while maintaining comparable accuracy, substantially reducing the computational cost of CDT construction (Fan and Roche, 2025).

Taken together, the Transformer model plays a pivotal role in the integration of cardiovascular multimodal data and the construction of CDT. It not only shows potential for enhancing the accuracy of diagnosis and prediction in cardiovascular diseases but also opens new avenues for future precision medicine and personalized therapy. However, current Transformer-based anatomical, generative, and surrogate models remain largely at the proof-of-concept stage and should not be interpreted as clinically validated cardiac digital twins.

### 3.5 Large language models (LLMs)

Large language models (LLMs) are gradually emerging as transformative tools in cardiovascular medicine, with applications spanning clinical decision support, medical documentation automation, patient communication, and research assistance (Quer and Topol, 2024; Ferreira Santos et al., 2025). LLMs are predominantly language-oriented foundation models that are commonly built on Transformer architectures and trained at scale on large text corpora. However, it is important to note that LLMs are not merely equivalent to the Transformer, but represent an evolution built upon its foundations, enhanced by advanced AI innovations such as instruction tuning (Naveed et al., 2025) whereas retrieval-augmented generation (RAG) is a deployment approach that supplements the model with information retrieved from external knowledge sources rather than an intrinsic capability of the underlying architecture (Lewis et al., 2020). Meanwhile, the rapid advancement of GPU computing power has provided crucial support for the evolution of LLMs. Collectively, these factors underpin the remarkable capabilities that define modern LLMs.

LLMs have demonstrated unique value in clinical decision support by integrating medical guidelines, clinical practice standards, the latest research evidence, and patient-specific medical records to provide evidence-based support for clinicians, thereby assisting in differential diagnosis, risk assessment, and treatment planning (Maity and Saikia, 2025). However, access to current guidelines and newly published evidence requires an up-to-date external knowledge source or retrieval mechanism. Compared with traditional decision support systems that rely heavily on clinicians' experience, multimodal LLMs can integrate textual information with additional data modalities, whereas conventional LLMs primarily operate on textual or text-converted clinical information (AlSaad et al., 2024; Nam et al., 2025). In cardiovascular medicine, the potential of LLMs has been validated in several studies. For instance, Wright et al. employed ChatGPT-4 to automatically extract real patient data from EHRs, calculate HEART scores, and predict 60-day major adverse cardiac events (MACE). The results showed that the HEART scores computed by ChatGPT-4 were on average higher than those determined by clinicians, while its predictive performance for MACE was comparable (Wright et al., 2025). Similarly, Kaya et al. (2024) reported that ChatGPT-4 achieved 83% accuracy for myocarditis assessment based on multicenter CMR reports together with available clinical and laboratory information, which was comparable to a radiologist with 1 year of CMR experience but lower than radiologists with 2 and 4 years of experience. More recently, a proof-of-concept study evaluating four LLMs for standardized CMR protocol generation reported moderate agreement with guideline-based reference protocols, with the best-performing model achieving 71.5% concordance (Licu et al., 2026). Complementing these findings, Terzis et al. retrospectively evaluated GPT-4-based sequence-level MRI protocol selection using 100 routine request forms across four subspecialties, including cardiac imaging. Overall protocol ratings were comparable to those of a junior radiology resident but lower than those of a more experienced resident, with performance varying across subspecialties (Terzis et al., 2026).

In medical documentation automation, LLMs have been widely applied to the automated generation of structured clinical records, including clinical note documentation, discharge summaries, and echocardiography reports. Although much of the current evidence on LLM-assisted documentation is cross-domain rather than cardiovascular-specific, multiple studies have shown that Ambient AI platforms based on LLMs can “listen” to clinician–patient interactions in real time and automatically generate clinical documentation, requiring only simple review and minor edits from clinicians. This process significantly reduces documentation time, improves same-day closure rates, and alleviates professional burnout caused by excessive clerical workload, enabling clinicians to devote more attention to patient care and communication (Olson et al., 2025). In addition, Ganzinger et al. (2025) employed LLMs to automatically generate German-language discharge summaries from structured clinical data, producing coherent and highly accurate drafts that serve as a valuable reference for research and practice in non-English medical documentation. Recent studies have further demonstrated that EchoGPT, a Llama-2–based model fine-tuned for echocardiography report summarization, can automatically generate draft reports for physician review and approval that were comparable to cardiologist-written reports in internal expert evaluation and were preferred for conciseness; however, performance decreased on an external echocardiography-report dataset. Such capabilities may help streamline the generation workflow of echocardiography reports, although further external and clinical validation is required (Chao et al., 2025).

As for patient communication, LLMs can translate complex medical terminology into plain, understandable language and provide actionable health education and tailored lifestyle guidance (Will et al., 2025; Aydin et al., 2024). In cardiovascular imaging, Salam et al. (2024) showed that GPT-4 substantially improved the readability and layperson-rated understandability of CMR reports while largely preserving factual correctness and completeness. However, the exploratory design and small layperson cohort limit the generalizability of these findings. Kozaily et al. (2024) found that both ChatGPT and Bard accurately and consistently answered 30 heart failure-related questions, suggesting the potential application of LLMs in heart failure education. Similarly, Lee et al. (2024) systematically evaluated ChatGPT's ability to address patient questions related to atrial fibrillation and found the responses accurate and easy to understand, underscoring its potential as a supplementary tool for patient engagement and follow-up. A recent bilingual evaluation of coronary heart disease patient education further showed that LLM performance varied according to both model and prompt language, emphasizing the need for language-specific validation before multilingual patient-facing deployment (Liu et al., 2026). Collectively, these findings suggest that LLMs may support patient communication and potentially reduce some communication burden; however, direct evidence that these applications reduce overall clinical workload remains limited.

In research assistance, LLMs excel at knowledge integration and evidence extraction, enabling rapid screening and analysis of vast bodies of literature, key information extraction, and evidence map construction, thereby markedly accelerating systematic reviews and meta-analyses (Luo X. et al., 2024). A previous study has demonstrated that LLMs can reduce reviewers' workload by 33%−93% in title- and abstract-level screening (Delgado-Chaves et al., 2025). Meanwhile, LLMs also exhibit remarkable accuracy in data extraction for evidence-based research, as a proof-of-concept study on Claude 2 demonstrated 96.3% accuracy across 160 data elements (Gartlehner et al., 2024).

Collectively, LLMs, represented by ChatGPT, are reshaping both clinical and research paradigms in cardiovascular medicine, not only enhancing workflow efficiency but also providing new momentum for the development of precision, personalized, and intelligent healthcare. More broadly, Transformer-based cardiovascular applications span diverse model families, data modalities, and clinical tasks, while their validation maturity and translational readiness remain heterogeneous (Figure 2).

**Figure 2:**
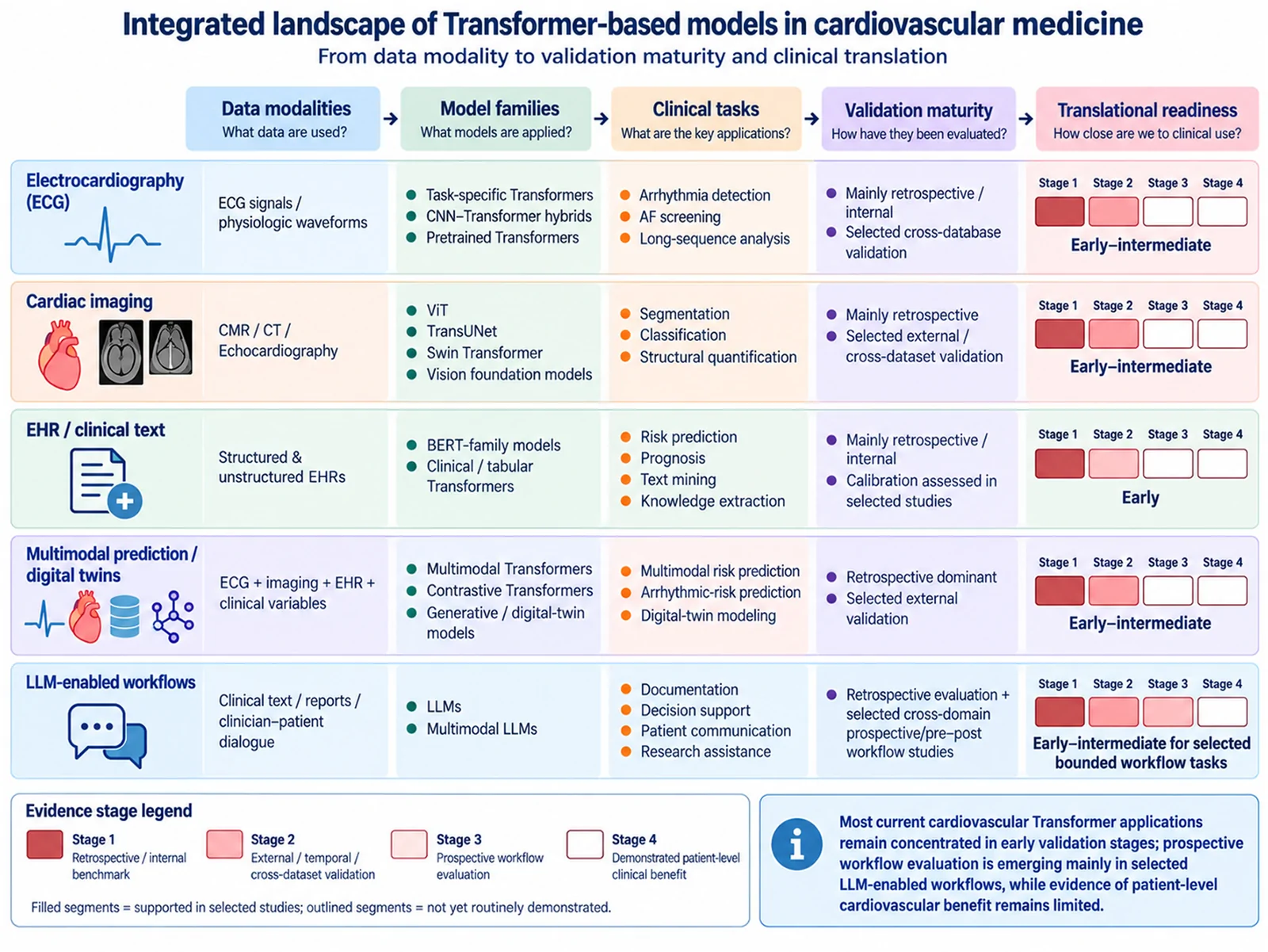
Integrated landscape of Transformer-based models in cardiovascular medicine from data modality to clinical translation. Created by the authors using BioRender.com, with AI-assisted visual design supported by OpenAI. ECG, electrocardiography; EHR, electronic health record; LLM, large language model. The figure provides a qualitative synthesis of representative evidence across model families, clinical tasks, and validation stages. The validation categories indicate general evidence maturity and should not be interpreted as demonstrating clinical readiness or patient-level benefit unless supported by prospective outcome studies.

## 4 Discussion

The studies summarized in this review show that Transformer-based methods have expanded from isolated proof-of-concept applications to a broad cardiovascular AI ecosystem encompassing ECG interpretation, cardiac imaging, EHR analysis, multimodal learning, and generative language applications (Vaid et al., 2023; Hu et al., 2022; Tudjarski et al., 2025; Alghieth, 2025; Busia et al., 2025; Zhao et al., 2022; Chen Z. et al., 2023; Alvén et al., 2025; Liao et al., 2023; Zhang et al., 2026; Chen et al., 2024; Fan et al., 2023; Liu et al., 2021; Cao et al., 2023; Yang et al., 2024; Zhang L. et al., 2025; Lee et al., 2020; Huang et al., 2019; Rasmy et al., 2021; Kim M. et al., 2024; Houssein et al., 2023; Antikainen et al., 2023; Kim et al., 2025; Xu et al., 2024; Cui et al., 2025; Turgut et al., 2025; Qiu et al., 2024; Girlanda et al., 2024; Lai C. et al., 2025; Corral-Acero et al., 2020; Sel et al., 2024; Thangaraj et al., 2024; Yu J. et al., 2025; Lai Y. et al., 2025; Fan and Roche, 2025). Their principal technical contribution is not a single task-specific performance gain, but a flexible representation-learning framework that can model long-range dependencies, exploit large-scale pre-training, and align heterogeneous data. Nevertheless, the available evidence does not establish that Transformers are universally superior to CNNs, RNNs, gradient-boosting methods, or carefully designed hybrid models. Relative performance remains dependent on the clinical task, sample size, signal structure, annotation quality, data preprocessing, and the strength of the comparator (Khan et al., 2023; Guo et al., 2021; Yang et al., 2022; Acosta et al., 2022; Lambert et al., 2024). The clinically relevant question is therefore not whether a model uses a Transformer architecture, but whether it delivers reproducible added value for a clearly defined decision, patient population, and care pathway.

### 4.1 Interpreting the contribution of Transformer-based models

Transformer, foundation model, multimodal foundation model, and large language model are related but non-equivalent concepts. A multimodal foundation model extends the foundation-model paradigm across multiple data modalities. A Transformer is an architecture based on attention mechanisms; a foundation model is a model trained at scale and adapted across multiple downstream tasks; and an LLM is a predominantly language-oriented foundation model that commonly uses a Transformer backbone (Vaswani et al., 2017; Nerella et al., 2024; Devlin et al., 2019; Brown et al., 2020; Dosovitskiy et al., 2020; Krittanawong et al., 2017; Cheplygina et al., 2019; Huang et al., 2023). Not every Transformer is a foundation model, and not every clinically useful cardiovascular foundation model is an LLM. This distinction is important because claims about general reasoning, multimodal competence, or transferability should be supported by the model's training objective and validation evidence rather than inferred from the architecture alone.

Similarly, end-to-end learning should be viewed as a design strategy rather than an inevitable successor to modular clinical AI. Joint optimization across raw inputs and clinical outputs may reduce hand-engineered interfaces and allow information to be shared across modalities. However, modular or hybrid systems can provide advantages in auditability, error localization, modality-specific validation, and fail-safe operation, particularly when data streams are intermittently unavailable or clinical risk is high (Acosta et al., 2022; Lambert et al., 2024; Feiner et al., 2023; Zhao et al., 2023; Lyu et al., 2022). A clinically defensible system may therefore combine a pre-trained Transformer encoder with validated task-specific heads, explicit uncertainty estimation, rule-based safety constraints, and clinician review. The appropriate balance between end-to-end and modular design should be determined by intended use, failure consequences, data availability, and the need for traceability—not by architectural novelty.

Evaluation should also move beyond headline discrimination metrics. For diagnostic and prognostic applications, studies should report calibration, confidence intervals, clinically meaningful operating points, subgroup performance, and added value over standard care and strong non-Transformer baselines. For workflow applications, relevant outcomes include time saved, false-alert burden, rate of corrected vs. introduced errors, clinician workload, downstream testing, and changes in treatment decisions. For generative systems, factuality, citation accuracy, consistency, and the ability to abstain or escalate uncertain cases are at least as important as linguistic fluency. These requirements are especially relevant to cardiovascular medicine, where small changes in threshold selection or risk calibration can alter triage, antithrombotic treatment, invasive testing, or long-term preventive therapy.

### 4.2 Barriers to generalizable and trustworthy clinical use

The first major barrier is generalizability. Many studies reviewed here use retrospective, curated, single-dataset evaluations. High performance under random internal splitting may not be preserved across hospitals, countries, demographic groups, device vendors, acquisition protocols, coding practices, or calendar periods (Guo et al., 2021; Yang et al., 2022). Cardiovascular data are particularly vulnerable to temporal and contextual shift because ECG hardware, imaging protocols, treatment patterns, and EHR documentation evolve over time. Patient-level separation, external and temporal validation, and transparent reporting of data provenance are therefore essential. Multimodal models introduce additional complexity: modalities are rarely collected synchronously, missingness may be clinically informative, and a high-dimensional modality can dominate the fused representation. Models should be tested under realistic missing-modality, delayed-input, and corrupted-signal scenarios rather than only when all inputs are complete (Acosta et al., 2022; Raminedi et al., 2024; Lymperaiou and Stamou, 2024).

The second barrier is reliability at the point of care. Transformer models may be poorly calibrated, overconfident outside the training distribution, or sensitive to subtle changes in input formatting. In multistage pipelines, uncertainty can propagate across segmentation, feature extraction, prediction, and reporting components (Lambert et al., 2024; Feiner et al., 2023). Uncertainty estimates should therefore be evaluated for calibration and linked to explicit actions such as requesting additional data, returning an indeterminate result, or escalating to specialist review. Interpretability also requires caution. Attention maps and *post hoc* feature-attribution methods can help generate hypotheses, but they do not by themselves establish causal reasoning or pathophysiological validity (Hassija et al., 2024). For LLM-based systems, plausible but unsupported statements and inaccurate references remain well-documented risks (Yu E. et al., 2025; Agarwal et al., 2026; Chelli et al., 2024). Retrieval augmentation, constrained outputs, source display, and mandatory human verification can reduce—but not eliminate—these risks.

The third barrier is the gap between technical performance and clinical benefit. Retrospective accuracy does not demonstrate that a model improves decisions, outcomes, efficiency, or equity when embedded in real workflows. The effect of an AI system depends on who receives its output, when it is delivered, how it is displayed, whether clinicians can override it, and whether it changes management. Poorly designed tools may increase alert fatigue, automation bias, or unnecessary testing even when their stand-alone accuracy is high. Prospective evaluation must therefore treat the model and its human-computer interface as a combined intervention. Early clinical studies should assess usability, safety, human factors, and failure modes before large-scale comparative trials (Vasey et al., 2022; Liu et al., 2020). Existing randomized trials of clinical AI are encouraging but remain concentrated in limited settings, reinforcing the need for multicenter studies and patient-relevant outcomes (Han et al., 2024).

The fourth barrier concerns governance across the model lifecycle. Cardiovascular AI requires access to sensitive longitudinal data, creating challenges related to consent, privacy, data linkage, cybersecurity, and cross-institutional sharing (Conduah et al., 2025; Zhang F. et al., 2025). Bias can arise from under-representation, differential access to testing, historical treatment patterns, and labels that encode prior clinical decisions; aggregate performance may therefore conceal clinically important subgroup harms (Chen R. J. et al., 2023). Governance should specify responsibility for erroneous outputs, version control, update procedures, audit trails, and thresholds for suspension or rollback. Resource-intensive model development may also widen disparities between well-resourced centers and institutions with limited data infrastructure. Federated or privacy-preserving approaches, lightweight adaptation, and evaluation in resource-constrained settings should be considered early rather than added after model development (Zhang F. et al., 2025).

Viewed across the different cardiovascular applications, translational evidence is uneven rather than uniformly absent. Cross-database or independent external validation has been demonstrated in selected ECG, cardiac imaging, multimodal, and LLM studies, whereas many other applications remain supported primarily by retrospective internal evaluation. Calibration has been explicitly assessed in selected EHR and multimodal models, and subgroup fairness analysis has been reported for some multimodal applications, but these practices are not yet routine across domains. Prospective workflow evaluation is emerging for LLM-enabled applications, whereas evidence of improvement in cardiovascular patient-level outcomes remains scarce. Table 2 summarizes these differences across the major application domains.

**Table 2:** Translational validation and evidence gaps across major cardiovascular applications of Transformer-based models.

Application domain	External/temporal validation	Calibration	Subgroup/fairness assessment	Dataset-shift/generalizability evidence	Prospective evaluation
ECG	Cross-database inter-patient evaluation was performed in the heart language model, whereas other representative ECG studies relied mainly on benchmark datasets (Hu et al., 2022; Tudjarski et al., 2025; Busia et al., 2025). Independent clinical-site and temporal validation remain limited	Probability calibration was not a primary evaluation endpoint in the representative ECG studies (Hu et al., 2022; Tudjarski et al., 2025; Busia et al., 2025)	Dedicated demographic subgroup/fairness analyses were not systematically reported in the representative ECG studies (Hu et al., 2022; Tudjarski et al., 2025; Busia et al., 2025)	Cross-database testing in the heart language model provides some evidence of generalization across ECG databases, but device-, vendor-, and clinical-site shifts were not specifically evaluated (Tudjarski et al., 2025)	No prospective clinical deployment or patient-outcome evaluation was identified in the representative ECG studies
Cardiac imaging	External validation was reported in selected studies, including an independent hospital cohort and evaluation across independent echocardiography datasets; temporal validation remains uncommon (Alvén et al., 2025; Zhang et al., 2026)	Probability calibration was not a primary evaluation endpoint in the cited imaging studies	Dedicated demographic fairness analyses were not systematically reported in the representative imaging studies	Selected studies provide some cross-site or cross-dataset evidence, but scanner-, vendor-, and protocol-related shifts have not been systematically evaluated (Alvén et al., 2025; Zhang et al., 2026)	Prospective clinical workflow or patient-outcome evaluation was not identified in the representative imaging studies
EHR/text mining	The acute myocardial infarction prognosis study used prospective multicenter registry data, but model evaluation was internal and the authors noted the absence of external-cohort validation (Kim M. et al., 2024). Antikainen et al. (2023) used a held-out test set within one healthcare system	The acute myocardial infarction prognosis study explicitly used confidence calibration, reliability diagrams, and expected calibration error (Kim M. et al., 2024); calibration was not routinely assessed across the other cited EHR applications	Dedicated demographic fairness evaluation was not identified in the representative EHR studies (Kim M. et al., 2024; Antikainen et al., 2023)	Direct dataset-shift experiments were not identified; temporal changes in coding, documentation, treatment patterns, and data availability remain important concerns	No prospective model deployment or clinical-impact evaluation was identified. Prospectively collected registry data in the AMI prognosis study do not constitute prospective evaluation of the AI intervention (Kim M. et al., 2024)
Multimodal models/cardiac digital twins	Lai C. et al. (2025) underwent independent external validation in a geographically separate healthcare system. The atrial fibrillation recurrence study used internal single-center validation, whereas UltraTwin used multicenter data with site-proportional splitting rather than an independent external cohort (Qiu et al., 2024; Yu J. et al., 2025)	Lai C. et al. (2025) reported class-balanced Brier scores in both internal and external validation, providing a calibration-related assessment of probabilistic predictions. Calibration was not a primary endpoint in the other representative multimodal/reconstruction studies	Lai C. et al. (2025) explicitly evaluated fairness across sex and age subgroups in the internal cohort; fairness analysis was not performed in the external cohort because of its limited size. Such analyses were not systematically reported in the other representative studies	Lai C. et al. (2025) was tested in an external cohort with differences in patient characteristics and available covariates, providing direct evidence of cross-site generalizability. Missing or asynchronous modalities remain an additional challenge for multimodal systems	UltraTwin was evaluated offline using multicenter data (Yu J. et al., 2025), while the AF-recurrence and MAARS studies were retrospective analyses (Qiu et al., 2024; Lai C. et al., 2025). Prospective clinical-impact evaluation remains lacking
LLM-enabled applications/ workflows	EchoGPT was validated at a geographically separate institutional site and on an independent external echocardiography-report dataset (Ganzinger et al., 2025). Kaya et al. (2024) used a multicenter retrospective dataset, while Wright et al. (2025) was a single-center retrospective study	Conventional probability calibration was not a principal evaluation approach in the representative generative/reporting studies; evaluation focused instead on task performance, factual correctness, agreement, and clinician-rated usefulness	Selected subgroup analyses were reported in the myocarditis study (Kaya et al., 2024), while exploratory clinician subgroup analyses were reported in a broader cross-domain ambient-AI study (Terzis et al., 2026); systematic patient-demographic fairness evaluation remains limited	EchoGPT showed a substantial performance decrease on an independent external report dataset with different report structure and writing style, indicating sensitivity to institutional/documentation shift (Ganzinger et al., 2025)	Workflow-oriented pre–post evaluation has been reported for ambient AI scribes (Terzis et al., 2026); however, this evidence is cross-domain rather than cardiovascular-specific and does not demonstrate improvement in cardiovascular patient-level outcomes

### 4.3 Priorities for clinical translation

A practical translation pathway should begin with a narrowly specified clinical problem: the intended user, target population, decision point, comparator, output, and anticipated consequence of error should be defined before model selection. The model should then be developed and reported using study-type-appropriate standards. TRIPOD+AI and PROBAST+AI support transparent reporting and assessment of prediction models; STARD-AI addresses AI-centered diagnostic accuracy studies; DECIDE-AI guides early-stage clinical evaluation; CONSORT-AI supports reporting of randomized AI intervention trials; and FUTURE-AI provides lifecycle-oriented principles for trustworthy deployment (Vasey et al., 2022; Liu et al., 2020; Collins et al., 2024; Lekadir et al., 2025; Sounderajah et al., 2025; Moons et al., 2025). These frameworks should be treated as complementary rather than interchangeable.

Validation should proceed in stages, with retrospective benchmark performance distinguished from evidence of generalizability and clinical utility. After internal development, investigators should perform independent external and temporal validation, stress testing, subgroup and fairness analyses, calibration assessment, and comparison with established clinical baselines. A prospective silent phase can then evaluate data availability, latency, distribution shift, and workflow fit without influencing care. However, prospective validation alone does not establish clinical utility. Limited live deployment should include predefined escalation and stopping rules, structured logging of overrides and errors, and qualitative assessment of clinician and patient experience. Only after these steps should comparative effectiveness studies evaluate whether the AI-assisted pathway improves clinical decision-making, resource use, patient experience, and clinically meaningful patient outcomes. Reporting should include the model version, preprocessing, input eligibility, handling of missing data, human-AI interaction, and analysis of failure cases so that findings can be reproduced and interpreted (Vasey et al., 2022; Liu et al., 2020; Han et al., 2024; Collins et al., 2024; Lekadir et al., 2025; Sounderajah et al., 2025; Moons et al., 2025).

For foundation models and LLMs, near-term deployment is most defensible when the task is bounded and verifiable, such as drafting documentation, extracting structured variables, retrieving evidence, or summarizing information for clinician review. Higher-risk functions—autonomous diagnosis, prognosis, or treatment recommendation—require calibrated uncertainty, traceable evidence, prospective validation, and a clearly accountable clinician in the loop (Hassija et al., 2024; Yu E. et al., 2025; Agarwal et al., 2026; Chelli et al., 2024; Singhal et al., 2023; Thirunavukarasu et al., 2023; Houssein et al., 2024). Model updates should be treated as changes to a medical intervention rather than routine software maintenance: performance should be monitored after deployment, with pre-specified drift indicators, periodic recalibration, versioned evaluation, and rollback capability (Lekadir et al., 2025). For cardiac digital twins, technical fidelity must be complemented by validation of the predicted physiological or therapeutic response; visually or statistically realistic generation alone is insufficient to establish clinical utility (Corral-Acero et al., 2020; Sel et al., 2024; Thangaraj et al., 2024; Yu J. et al., 2025; Lai Y. et al., 2025; Fan and Roche, 2025).

The most plausible future is therefore not unrestricted autonomous cardiovascular AI, but an adaptive human-AI partnership. Transformers may organize and synthesize complex data at a scale that is difficult for individual clinicians, while clinicians provide contextual interpretation, value-sensitive judgment, communication, and responsibility. Research should explicitly test how this partnership changes decisions and outcomes, including when clinicians appropriately reject the model. Such evidence will be more informative than demonstrations that an algorithm can match a specialist on an isolated benchmark.

### 4.4 Limitations of this review

This article is a narrative review. We used a structured literature-identification process and report the databases, search concepts, date range, study-selection principles, and handling of conference papers/preprints; however, the review did not use a preregistered systematic protocol, duplicate study selection, formal risk-of-bias assessment, or meta-analysis. The included literature spans heterogeneous tasks, cohorts, split strategies, endpoints, and metrics, so reported performance values are not directly comparable. Several emerging applications—particularly digital-twin engineering and some multimodal systems—remain supported by conference papers or preprints. Publication and selection bias may favor positive findings, and the review focuses on Transformer-related methods rather than exhaustively comparing every alternative statistical or machine-learning approach. The conclusions should therefore be interpreted as a critical narrative synthesis and translational research agenda rather than a quantitative estimate of clinical effectiveness.

## 5 Conclusions

Transformer-based models provide a flexible framework for representing longitudinal, multimodal, and unstructured cardiovascular data, and they have generated promising results across ECG analysis, cardiac imaging, EHR mining, multimodal prediction, digital twins, and language-enabled workflows. Architecture alone, however, is insufficient for clinical value. Translation requires robust external and temporal validation, calibration and uncertainty management, explicit comparison with standard care, safe human-AI interaction, prospective evidence of clinical utility, and lifecycle governance. In the near term, the strongest role for these models is likely to be carefully bounded decision support that augments clinicians rather than replaces them. Recent studies further emphasize that future cardiovascular AI should extend beyond isolated benchmark performance toward multimodal integration, foundation-model–enabled research workflows, improved generalizability, and clinically meaningful implementation (Yang et al., 2025; Xu et al., 2026). Progress should be judged not by model scale or benchmark performance alone, but by reproducible improvements in cardiovascular care, safety, efficiency, and equity.
